# Inhibitory Effect of Lotusine on Solar UV-Induced Matrix Metalloproteinase-1 Expression

**DOI:** 10.3390/plants11060773

**Published:** 2022-03-14

**Authors:** Tae-Kyeong Ryu, Eunmiri Roh, Han-Seung Shin, Jong-Eun Kim

**Affiliations:** 1 Department of Food Science and Biotechnology, Dongguk University-Seoul, Goyang-si 10326, Korea; tk1577@naver.com; 2 Department of Cosmetic Science, Kwangju Women’s University, Gwangju 62396, Korea; roheunmiri@kwu.ac.kr; 3 Department of Food Science and Tecshnology, Korea National University of Transportation, Chungju-si 27909, Korea

**Keywords:** lotusine, anti-wrinkle, phytochemical, MMP-1, ultraviolet

## Abstract

Solar ultraviolet (sUV) radiation remains a major cause of skin aging. *Nelumbo nucifera* (lotus) is a well-known edible plant widely grown in Asia, including Korea, China, and Japan. The lotus consists of flowers, leaves, stems, and seeds, and all parts reportedly possess nutritional and medical values. Traditionally, lotus flowers, leaves, stems, and seeds have been used as antidiarrheal agents, diuretics, antipyretics, and antimicrobial and antihyperlipidemic agents. In addition, the *Nelumbo nucifera* lotus embryo has been shown to possess sedative and antipyretic properties and can relieve hemostatic thirst and treat eye diseases. Recently, *Nelumbo nucifera* lotus flower extract has been widely used in cosmetics due to its ability to reduce wrinkles and its whitening effects. Numerous cosmetics using *Nelumbo nucifera* lotus embryo extracts are commercially available. However, the active components of *Nelumbo nucifera* remain elusive. Lotusine is a phytochemical and soluble alkaloid found in lotus embryos. Herein, we examined the anti-wrinkle effect of lotusine using sUV-exposed human keratinocytes. We observed that lotusine reduced sUV-induced matrix metalloproteinase (MMP)-1 expression and modulated transcriptional activities of activator protein (AP)-1 and nuclear factor kappa B (NF-κB). sUV-induced AP-1 and NF-κB activity could be activated via multiple signal transduction cascades, including the p38 MAPK, JNK, ERK1/2, and Akt pathways in the skin. Lotusine inhibited the MEK1/2-ERK1/2-p90^RSK^, MKK3/6-p38, and Akt-p70^S6K^ pathways. Overall, our findings suggest that lotusine has potential benefits related to MMP-1 expression and skin aging following sUV exposure. Hence, the lotus can be developed as a valuable functional food and cosmetic material.

## 1. Introduction

The skin is in direct contact with the environment and therefore undergoes aging owing to environmental damage. Solar ultraviolet (sUV) irradiation has been implicated as the primary environmental factor causing skin aging in humans [1]. Reportedly, sUV has deleterious effects on human skin, including sunburn, immune suppression, cancer, and premature aging [2]. The sun emits UV radiation across a broad spectrum, ranging from the high-energy UVC (wavelengths ˂280 nm) and UVB (280–315 nm) bands to the UVA band (315–400 nm) [3]. UVC is effectively blocked from reaching the surface of the Earth by the stratospheric ozone layer, although accidental exposure could occur from human-made sources, such as germicidal lamps. Nevertheless, UVA and UVB radiation both reach the earth’s surface in sufficient amounts to induce important biological consequences on the skin and eyes [4]. Of the UV light reaching the earth’s surface, approximately 5% is UVB, while the remaining 95% is UVA [5]. These two types of UV radiations can both cause skin aging [6].

sUV reportedly induces the expression of matrix metalloproteinases (MMPs) that degrade skin collagen and other extracellular matrix (ECM) proteins [7]. Repeated exposure to sUV radiation can stimulate ECM protein breakdown, fragmentation, and organized collagen and inhibits procollagen biosynthesis owing to MMP overexpression, resulting in collagen loss [1]. Certain MMPs, including MMP-1, MMP-2, and MMP-9, are primarily expressed in the skin [2]. Among these, MMP-1 has been shown to initiate the degradation of type I and III fibrillar collagens, which comprise the dermal ECM. Thus, reducing collagen degradation by inhibiting MMP-1 expression could prevent wrinkles, thus representing a potential therapeutic strategy for photoaging [8].

Transcription factors such as activator protein (AP)-1 and nuclear factor kappa B (NF-κB) are known to be associated with skin aging, additionally regulating signal transduction pathways involved in the modulation of UV-induced MMP-1 expression [9]. AP-1 is a heterodimer that plays an important role in several inflammatory processes [10]. NF-κB regulates immune responses and inflammation [11]. UV-induced AP-1 and NF-κB activity is known to activate multiple signal transduction cascades, such as the p38 mitogen-activated protein kinase (MAPK), JNK, ERK1/2, and Akt pathways, in skin cells. Thus, regulation of these pathways has been employed as a strategy to suppress UV-induced MMP-1 expression [12].

*Nelumbo nucifera* is a well-known edible plant widely grown in Korea, China, and Japan [13]. *Nelumbo nucifera* is composed of flowers, leaves, stems, and seeds. Notably, all lotus organs have nutritional and medical values and are deemed specific foods and herbs [14]. Traditionally, lotus flowers, leaves, stems, and seeds have been used as antidiarrheals, diuretics, antipyretics, and antimicrobial and antihyperlipidemic agents [15]. In addition, the *Nelumbo nucifera* lotus embryo has been used as a sedative and antipyretic, as well as for hemostatic thirst relief and treatment of eye diseases [16]. Recently, *Nelumbo nucifera* lotus flower extract has been widely used in cosmetic preparations owing to its ability to reduce wrinkles and its whitening properties [17]. Currently, several cosmetics using *Nelumbo nucifera* lotus embryo extracts are commercially available [13]. However, the active components of lotus embryos need to be elucidated.

**Figure 1:**
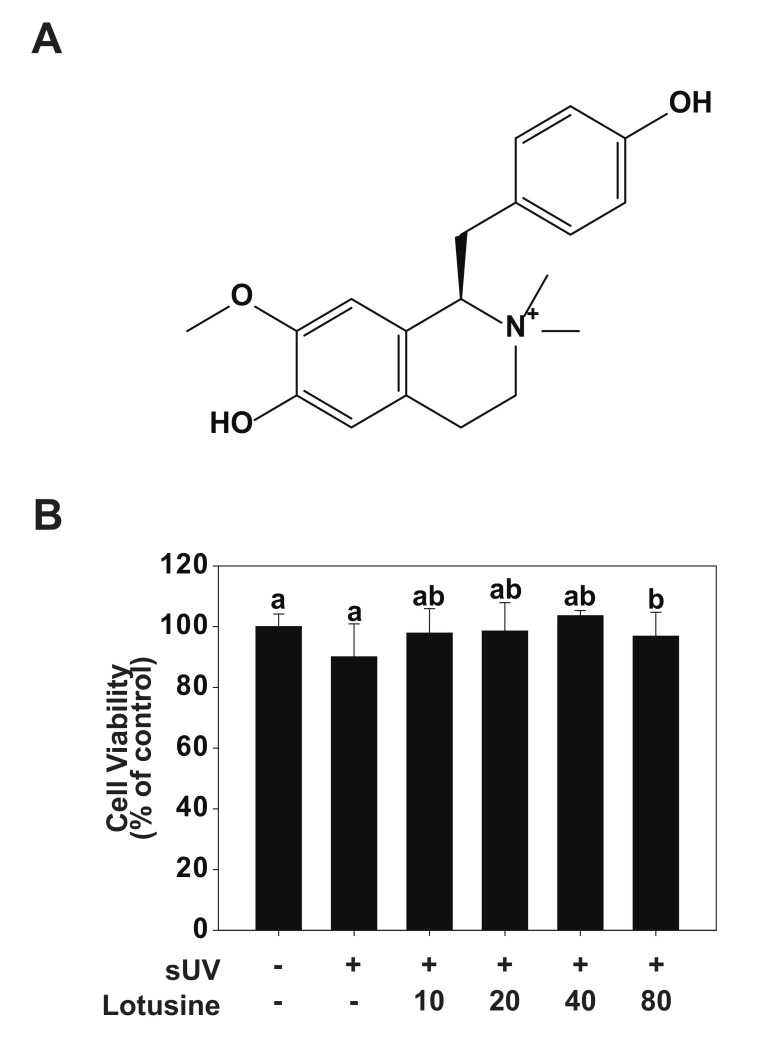
Effect of lotusine on HaCaT cell viability. (**A**) Chemical structure of lotusine. (**B**) MTT assay results showed that lotusine does not exhibit cytotoxicity up to a concentration of 80 µM. Means with different letters (a,b) within a graph significantly differ from each other at *p* < 0.05.

Lotusine (Figure 1A) is a phytochemical and soluble alkaloid found in lotus embryos [18]. Previous studies have shown that lotusine possesses antihypertensive, antibacterial [19], anti-inflammatory, and hypoglycemic activities [20]. In addition, lotusine has shown sedative and anticonvulsant properties and can benefit the heart, eliminating pathogenic fever and spontaneous hemorrhage [21,22]. However, the anti-aging effects of lotusine and its underlying molecular mechanisms must be established. In the present study, we revealed the effect of lotusine on UV-induced MMP-1 expression in cultured human keratinocytes (HaCaT cells) and determined the underlying molecular mechanisms.

## 2. Results

### 2.1. Cytotoxicity of Lotusine 

To validate the effect of lotusine on HaCaT cells, cell viability was evaluated following sUV irradiation. Lotusine treatment was applied after sUV irradiation, followed by the MTT assay. In the MTT assay, cell viability of ≥80% is deemed non-cytotoxic, 80–60% is considered weakly cytotoxic, and ˂60–40% and 40% is considered strongly cytotoxic [23]. Based on the MTT results, a maximum concentration of 80 μM lotusine showed a cell survival rate of 97%, which was 100% at the minimum concentration assessed (10 μM). These findings indicated that lotusine did not affect the survival of HaCaT cells (Figure 1B). 

### 2.2. Effects of Lotusine on sUV-Induced MMP-1 Expression in HaCaTCcells 

**Figure 2:**
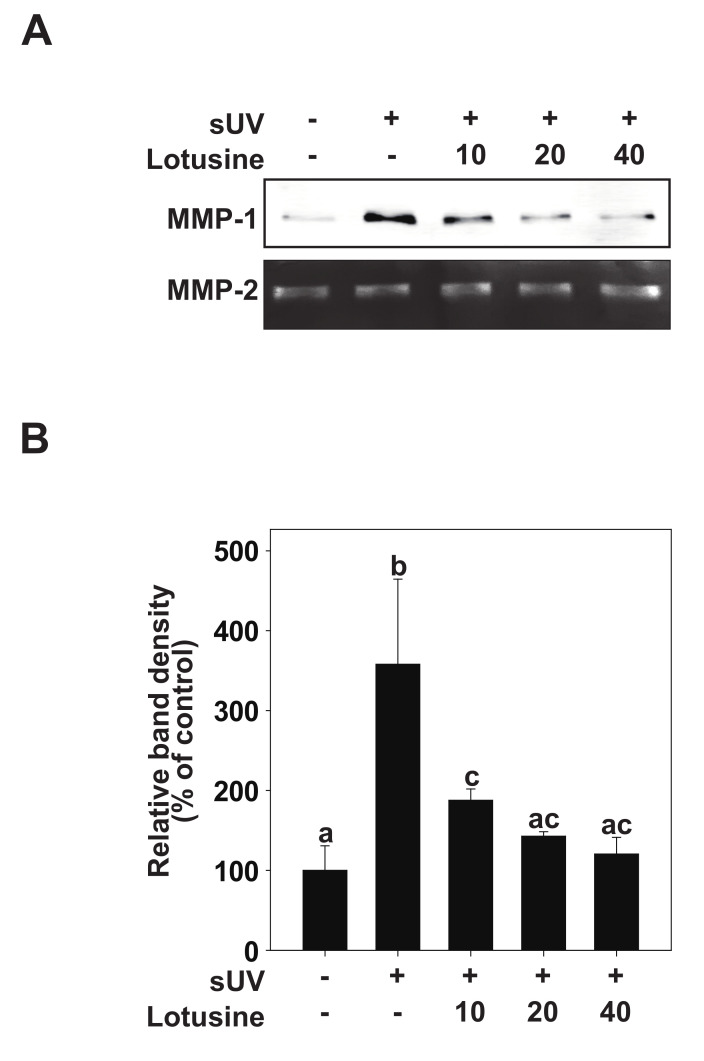
Lotusine inhibits solar ultraviolet (sUV)-induced MMP-1 protein expression in HaCaT cells. (**A**) HaCaT cells were pretreated with lotusine at indicated concentrations for 1 h and further treated with 25 kJ/m^2^ sUV for 48 h at 37 °C. (**B**) MMP-1 protein expression data for HaCaT cells were quantified using Image Studio software (LI-COR, NE). Data (*n* = 3) are presented as mean ± standard deviation (SD). Bars marked with different letters (a–c) significantly differ (*p* < 0.05) according to Duncan’s multiple range test. MMP-1, matrix metalloproteinase-1.

It has been suggested that sUV-exposed skin cells (fibroblasts, keratinocytes) can demonstrate aging biomarkers such as DNA damage and cell cycle arrest, potentially elevated due to excessive MMP secretion [8]. sUV induces the synthesis and expression of MMP-1 in the human epidermis, which breaks down fibrin collagen and plays an important role in photoaging [9]. To determine the inhibitory effect of lotusine on MMP-1, the inhibitory effect on sUV-induced MMP-1 protein expression was evaluated in HaCaT cells. sUV irradiation (25 kJ/m^2^) was performed after pretreatment with lotusine. After 48 h, MMP-1 protein secreted from the culture medium was analyzed by Western blotting. These results revealed that MMP-1 protein expression increases following sUV irradiation, and lotusine decreased MMP-1 expression. MMP-2 was used as a loading control (Figure 2A,B).

### 2.3. Effects of Lotusine on sUV-Induced MMP-1 Promoter Activity by Suppressing AP-1 and NF-κB Transactivation

**Figure 3:**
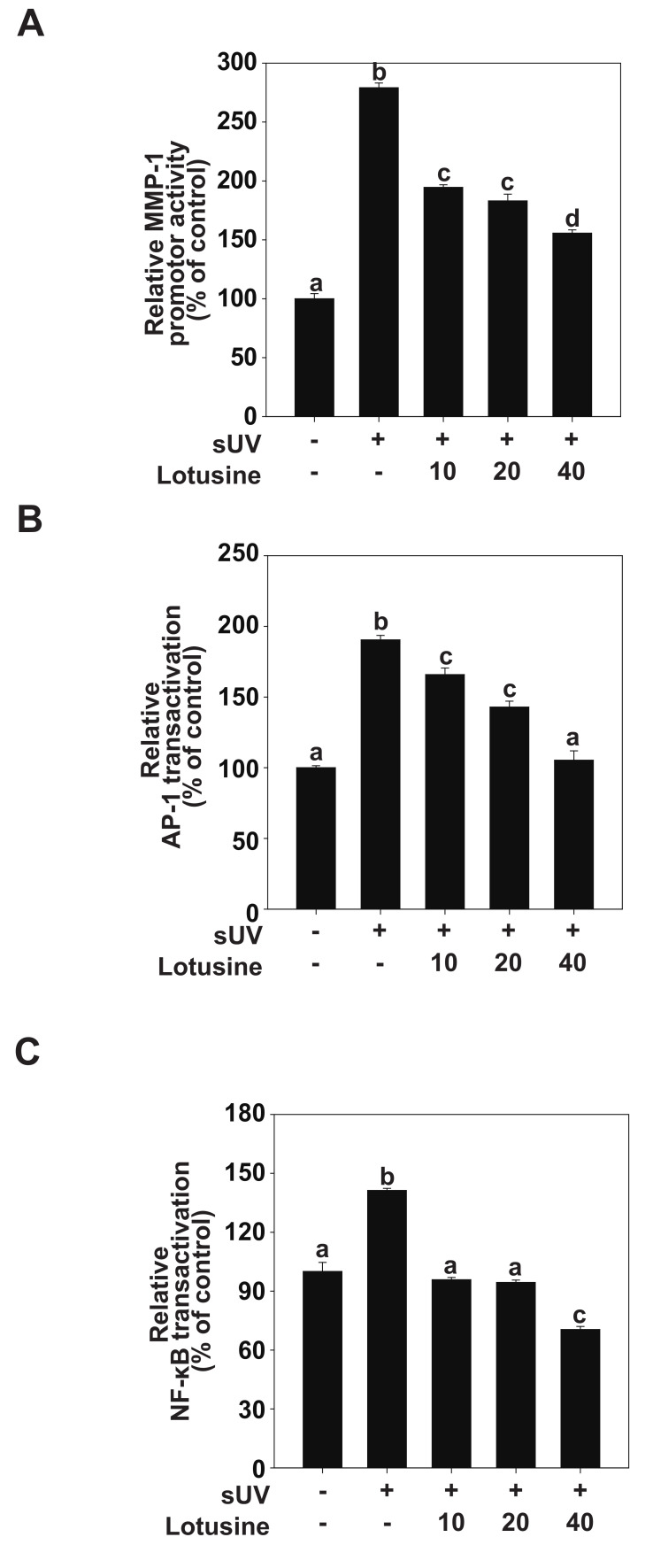
Lotusine suppresses solar ultraviolet (sUV)-induced MMP-1 transcription by inhibiting AP-1 and NF-κB transactivation in HaCaT cells. (**A**) MMP-1 promoter activity was measured using the luciferase assay. Transactivation of AP-1 (**B**) and NF-κB (**C**) was measured using the luciferase assay. Bars marked with different letters (a–d) significantly differ (*p* < 0.05) according to Duncan’s multiple range test. MMP-1, matrix metalloproteinase-1; AP-1, activator protein; NF-κB, nuclear factor kappa B.

AP-1 and NF-κB are major transcription factors involved in UV-induced MMP-1 expression [24]. We evaluated MMP-1 transcriptional activity and transactivation of AP-1 and NF-κB to clarify how lotusine regulates MMP-1 expression following sUV irradiation. The luciferase reporter gene analysis revealed that sUV irradiation increased the MMP-1 promoter activity level by 279% (Figure 3A). Treatment with lotusine decreased sUV-induced MMP-1 transcriptional activity. sUV-induced AP-1 transactivation increased by 190%, and treatment with 40 µM lotusine decreased this activity when compared with the untreated control (Figure 3B). The sUV-induced NF-κB transactivation was increased by 141%; treatment with 40 µM lotusine decreased this activity by 70% (Figure 3C). Therefore, we postulate that lotusine inhibited sUV-induced MMP-1 transcription by inhibiting NF-κB and AP-1 transactivation.

### 2.4. Lotusine Suppresses sUV-Induced MEK1/2-ERK1/2-p90^RSK^, MKK3/6-p38 and Akt-p70^S6K^ Pathways in HaCaT Cells 

**Figure 4:**
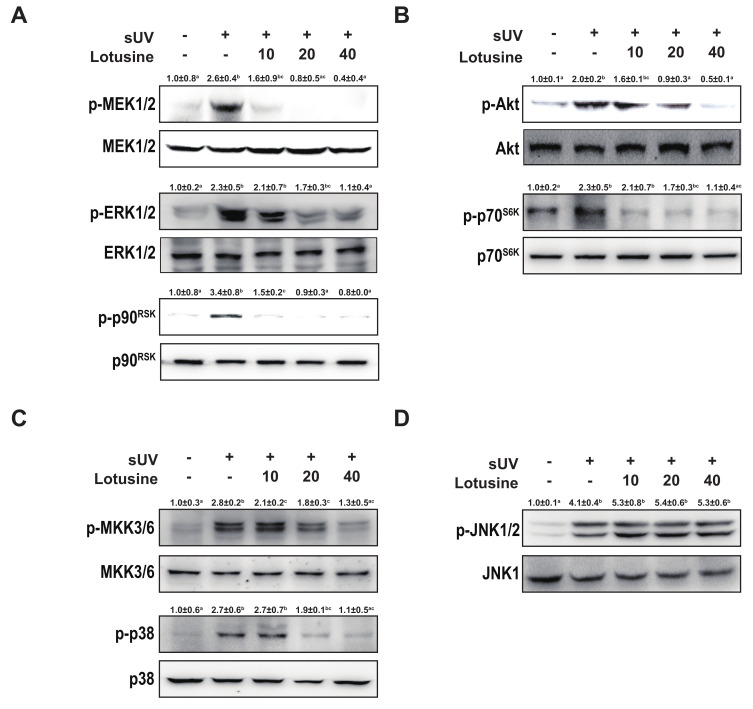
Lotusine suppresses solar ultraviolet (sUV)-induced MEK1/2-ERK1/2-p90^RSK^, MKK3/6-p38, and Akt-p70^S6K^ pathways in HaCaT cells. (**A**) and (**B**) Effect of lotusine on sUV-induced phosphorylation of the MEK1/2-ERK1/2-p90^RSK^ and Akt-p70^S6K^ signaling pathway. (**C**) and (**D**) Effect of lotusine on sUV-induced phosphorylation of the MKK3/6-p38 and JNK1/2 signaling pathways. Phosphorylation and total protein levels were analyzed by Western blotting. Relative band strength is indicated by the folding of the control and at the top of each band using Image Studio software (LI-COR). Different letters (a–d) significantly differ (*p* < 0.05) according to Duncan’s multiple range test.

Activating the MAPK and Akt pathways can activate transcription factors AP-1 and NF-κB [25]. Therefore, we investigated MAPK and Akt pathways. sUV irradiation significantly increased the phosphorylation levels of MEK1/2-ERK1/2-p90^RSK^, MKK3/6-p38, JNK1/2, and Akt-p70^S6K^. Treatment with lotusine inhibited the phosphorylation of MEK1/2-ERK1/2-p90^RSK^, MKK3/6-p38, and Akt-p70^S6K^ (Figure 4A–C). However, lotusine did not regulate the phosphorylation of JNK1/2 (Figure 4D). Therefore, lotusine inhibited MMP-1 expression via the MEK1/2-ERK1/2-p90^RSK^, MKK3/6-p38, and Akt-p70^S6K^ pathways.

## 3. Discussion

*Nelumbo nucifera* has been employed for edible or medicinal purposes, using various parts such as flowers, leaves, stems, and seeds [13]. Since ancient times, it has been used as an obstipatic, diuretic, antipyretic, and antibacterial agent. The lotus embryo reportedly contains alkaloids such as lotusine, neferine, roemerin, and nuciferine [26]. Lotusine, a phytochemical and soluble alkaloid, has been shown to dilate blood vessels and reduce blood pressure. In addition, lotusine can reduce intestinal motility and relieve diarrhea and has shown antispasmodic and beneficial cardiac properties [13]. It also eliminates pathogenic fever and spontaneous bleeding from the heart [26]. However, data on lotusine-mediated improvement in skin health are lacking. In the present study, we investigated the effects of lotusine on sUV-induced photoaging.

The skin is increasingly exposed to UV irradiation, causing photoaging, and the risk of photooxidation damage increases with long-term harmful effects [27]. Skin photoaging is an aging process typically associated with symptoms such as wrinkles, sagging, and loss of elasticity [8]. sUV irradiation induces the overexpression of MMP-1, which plays a major role in wrinkle formation by damaging tissue integrity [12]. MMPs are a family of structurally related matrix-degrading enzymes that play a critical role in various destructive processes, including inflammation, tumor intrusion, and skin aging [28]. UV-induced MMP-1 initiates collagen destruction by cleaving fibrillar collagen (types I and III) at a single cleavage site [27]. UV-radiation-induced activation of cell surface receptors stimulates the signaling pathways of MAPK family members, including ERK, JNK, and p38, which induce activation of AP-1 and NF-κB transcription factors, subsequently enhancing MMP-1 expression [27].

Herein, lotusine effectively inhibited sUV-induced MMP-1 expression and MMP-1 transcription in human keratinocytes. AP-1 and NF-κB transactivation was increased following sUV irradiation. Lotusine significantly inhibited MMP-1 promoter activity. In addition, lotusine significantly inhibited sUV-induced phosphorylation of the MEK1/2-ERK1/2-p90RSK, MKK3/6-p38, and Akt-p70S6K pathways. However, JNK phosphorylation was unaltered. Many phytochemicals directly inhibit the activity of kinases and thus regulate the signaling system. Lotusine inhibited the activity of the upper proteins, ERK1/2, p38, and Akt. However, it did not regulate the activity of the upper enzyme of JNK1/2. Thus, lotusine could potentially mediate its effects through single or multiple direct molecular targets. Among the sUV-induced signaling pathways, it is possible to regulate specific proteins that control all pathways, except JNK1/2. Furthermore, sUV might regulate several proteins, given the lotusine-mediated effects. Further research should be conducted to identify the direct molecular targets.

This study revealed the potential of lotusine for developing skin anti-aging agents. We observed that lotusine inhibited MMP-1 protein expression at non-cytotoxic concentrations. In most studies, UVA and UVB have been independently investigated [12]. However, using the same ratio of UV wavelengths as that irradiated by the sun is recommended for a more accurate physiological relevance. Therefore, we used a lamp capable of simulating the sun. The lamp emits UV light, consisting of 94.5% UVA and 5.5% UVB, and we used 25 kJ/m^2^ in the present study. This dose reportedly corresponds to the amount of UV available for approximately 1 h in Seoul (Korea) during the month of April [29].

In conclusion, we demonstrated the potential anti-aging effects of lotusine. Lotusine exhibits inhibitory activity against sUV-induced MMP-1 expression in HaCaT cells. In addition, lotusine inhibited sUV-induced MMP-1 transcription by suppressing AP-1 and NF-κB transactivation, thus regulating the MEK1/2-ERK1/2-p90^RSK^, MKK3/6-p38, and Akt-p70^S6K^ pathways. Thus, lotusine can be developed as a potential novel anti-wrinkle agent in cosmetic formulations to prevent photoaging.

## 4. Materials and Methods

### 4.1. Chemicals and Reagents

Lotusine (98.63%) was purchased from Indofine Chemical (Hillsborough, NC, USA). Dulbecco’s modified Eagle medium (DMEM) and penicillin–streptomycin solution were purchased from Welgene (Gyeongsan, Korea). Fetal bovine serum (FBS) was purchased from Atlas Biologicals (Fort Collins, CO, USA). The MMP-1 antibody was purchased from R&D Systems Inc. (Minneapolis, MN, USA). Antibodies against phosphorylated extracellular signal-regulated kinase (ERK) 1/2 at the Thr202/Tyr204 residues, total ERK1/2, total Akt, and total c-Jun N-terminal kinase 1 (JNK1) were procured from Santa Cruz Biotechnology (Santa Cruz, CA, USA). Other antibodies were purchased from Cell Signaling Biotechnology (Beverly, MA, USA). Lentiviral expression vectors including pGF-AP1-mCMV-EF1-Puro, pGF-NF-κB-mCMV-EF1-Puro (System Biosciences, Palo Alto, CA, USA), and pGF-MMP-1-mCMV-EF1-puro vector were kindly provided by Dr. Sung-Keun Jung (Korea Food Research Institute, Sung-Nam, Korea). These MMP-1 promoter vectors were cloned to the pGF vectors [30]. Packaging vectors (pMD2.0G and psPAX) were purchased from Addgene Inc. (Cambridge, MA, USA). A chemiluminescence detection kit was purchased from Thermo Fisher Scientific (Waltham, MA, USA).

### 4.2. Cell Culture and Viability 

HaCaT cells were purchased from CLS Cell Lines Services GmbH (Heidelberg, Germany) and cultured in DMEM supplemented with 10% FBS and 1% penicillin/streptomycin at 37 °C and 5% CO_2_. The MTT assay was employed to evaluate the cytotoxicity of lotusine. HaCaT cells were cultured in 96-well plates and treated with lotusine diluted in serum-free media. After 48 h, the cells were treated with MTT solution (0.5 mg/mL) and incubated at 37 °C for 4 h. Dimethyl sulfoxide (DMSO; 100 μL) was then added to each well to dissolve the formazan crystals, and absorbance was measured at 570 nm.

### 4.3. sUV Irradiation 

UVA-340 lamps (Q-Lab Corporation, Cleveland, OH, USA) produced optimal sunlight simulation, with a peak emission of 340 nm in the critical short wavelength region from 365 nm to 295 nm. The percentage of UVA and UVB produced by the UVA-340 lamps was measured using a UV meter at 94.5% and 5.5%, respectively. HaCaT cells were irradiated with sUV radiation (25 kJ/m^2^) in serum-free media.

### 4.4. Western Blotting

Cultured HaCaT cells were incubated in serum-free DMEM for 24 h and then treated with 10, 20, or 40 μM of lotusine for 1 h, followed by sUV irradiation. For MMP-1, the medium was harvested on ice and centrifuged at 15,000× *g* for 15 min. For protein extraction, cell lysates were prepared using cell lysis buffer (50 mM Tris-HCl at pH 8.0, containing 0.15 M NaCl, 1% NP-40, 0.1% sodium dodecyl sulfate (SDS), 0.5% deoxycholate, 1 mM dithiothreitol, 1 mM phenylmethylsulfonyl fluoride, and 1mM sodium vanadate). The protein concentration was determined using a protein analysis kit (Bio-Rad, Hercules, CA, USA). Proteins were electrophoretically separated using a 10% SDS-polyacrylamide gel and subsequently transferred onto polyvinylidene fluoride membranes (Merck Millipore, Burlington, MA, USA). After blotting, the membranes were blocked with 5% skim milk in 10% phosphate-buffered saline for 1 h. The membranes were incubated overnight with specific primary antibodies at 4 °C. The protein bands were visualized using an ECL substrate kit (Thermo Fisher Scientific, Waltham, MA, USA) after the addition of horseradish peroxidase (HRP)-conjugated secondary antibodies (Santa Cruz, CA, USA). Western blot data were analyzed using quantitative analysis in the Image Studio software (LI-COR, Lincoln, NE, USA).

### 4.5. Gelatin Zymography 

Gelatin zymography was used to assess MMP-2 expression. Briefly, protein samples were mixed with loading buffer (10% SDS, 0.25 M Tris buffer at pH 6.8, containing 25% glycerol and 0.1% bromophenol blue), incubated for 15 min at room temperature, and subjected to 10% SDS-polyacrylamide gel in the presence of 0.1% gelatin (*w*/*v*). Then, the gels were washed with renaturing buffer twice for 20 min and incubated for 48 h at 37 °C in developing buffer. After 48 h, the gel was stained with 0.5% Coomassie Brilliant Blue.

### 4.6. Luciferase Reporter Gene Assay 

pGF-AP-1-mCMV-EF1-Puro vector, pGF-NF-κB-mCMV-EF1-Puro, and pGF-MMP-1-mCMV-EF1-puro vector, with the packaging vectors (psPAX and pMD2.0G), were transfected into HEK293T cells using jetPEI, following the manufacturer’s instructions. The transfection medium was replaced 24 h after transfection. Then, cells were cultured for another 36 h, and viral particles were prepared using a syringe filter (0.45 μm). HaCaT cells were infected with 8 μg/mL polybrene (EMD Millipore, Burlington, MA, USA) overnight. The cell culture medium was replaced with fresh growth medium, and the cells were allowed to recover for 24 h before selection with 2 mg/mL puromycin (Sigma, Saint Louis, MO, USA) for 36 h. Next, HaCaT cells were starved in serum-free media for 24 h. Then, the cells were treated with lotusine for 1 h, followed by sUV irradiation. After either 12 h (AP-1) or 24 h (NF-κB, MMP-1), cell extracts were prepared using reporter lysis buffer (Promega, Madison, MA, USA). Transactivation was measured using a luciferase reporter gene analysis kit (Promega, Madison, MA, USA).

### 4.7. Statistical Analysis 

All experiments were performed in triplicate, and data were analyzed using SPSS Statistics software (version 23.0; IBM Co., New York, NY, USA). Differences between groups were compared using Duncan’s multiple range test and one-way ANOVA. A *p*-value ˂ 0.05 was deemed statistically significant.
